# Survival Analysis of Training Methodologies and Other Risk Factors for Musculoskeletal Injury in 2-Year-Old Thoroughbred Racehorses in Queensland, Australia

**DOI:** 10.3389/fvets.2021.698298

**Published:** 2021-11-02

**Authors:** Kylie L. Crawford, Anna Finnane, Ristan M. Greer, Tamsin S. Barnes, Clive J. C. Phillips, Solomon M. Woldeyohannes, Emma L. Bishop, Nigel R. Perkins, Benjamin J. Ahern

**Affiliations:** ^1^School of Veterinary Science, The University of Queensland, Gatton, QLD, Australia; ^2^School of Public Health, The University of Queensland, Herston, QLD, Australia; ^3^Torus Research, Bridgeman Downs, QLD, Australia; ^4^Mater Research Institute, The University of Queensland, Brisbane, QLD, Australia; ^5^Queensland Alliance for Agriculture and Food Innovation, The University of Queensland, Gatton, QLD, Australia; ^6^Curtin University Sustainability Policy (CUSP) Institute, Curtin University, Perth, WA, Australia; ^7^Garrards Equine Veterinary Practice, Albion, QLD, Australia

**Keywords:** racehorse, training, epidemiology, 2-year-old, musculoskeletal injury, causal model, Weibull, time-varying

## Abstract

Musculoskeletal injuries remain a global problem for the Thoroughbred racing industry and there is conflicting evidence regarding the effect of age on the incidence of injuries. The ideal time to commence race training is strongly debated, with limited supporting literature. There is also conflicting evidence regarding the effect of high-speed exercise on musculoskeletal injuries. There is a strong interest in developing training and management strategies to reduce the frequency of injuries. The types of musculoskeletal injuries vary between 2-year-old and older horses, with dorsal metacarpal disease the most common injury in 2-year-old horses. It is likely that risk factors for injury in 2-year-old horses are different than those for older horses. It is also likely that the risk factors may vary between types of injury. This study aimed to determine the risk factors for musculoskeletal injuries and dorsal metacarpal disease. We report the findings of a large scale, prospective observational study of 2-year-old horses in Queensland, Australia. Data were collected weekly for 56-weeks, from 26 trainers, involving 535 2-year-old Thoroughbred racehorses, 1, 258 training preparations and 7, 512-weeks of exercise data. A causal approach was used to develop our statistical models, to build on the existing literature surrounding injury risk, by incorporating the previously established causal links into our analyses. Where previous data were not available, industry experts were consulted. Survival analyses were performed using Cox proportional hazards or Weibull regression models. Analysis of musculoskeletal injuries overall revealed the hazard was reduced with increased exposure to high-speed exercise [Hazard ratio (HR) 0.89, 95% Confidence Interval (CI) 0.84, 0.94, *p* < 0.001], increased number of training preparations (HR 0.58, 95% CI 0.50, 0.67, *p* < 0.001), increased rest before the training preparation (HR 0.89, 95% CI 0.83, 0.96, *p* = 0.003) and increased dam parity (HR 0.86, 95% CI 0.77, 0.97, *p* = 0.01). The hazard of injury was increased with increasing age that training commenced (HR 1.13, 95% CI 1.06, 1.19, *p* < 0.001). Analyses were then repeated with the outcome of interest dorsal metacarpal disease. Factors that were protective against dorsal metacarpal disease and musculoskeletal injuries overall included: increased total cumulative distance (HR 0.89, 95% CI 0.82, 0.97, *p* = 0.001) and total cumulative days exercised as a gallop (HR 0.96, 95% CI 0.92, 0.99, *p* = 0.03), the number of the training preparations (HR 0.43, 95% CI 0.30, 0.61, *p* < 0.001). The age that training commenced was harmful for both dorsal metacarpal disease (HR 1.17, 95% CI 1.07, 1.28, *p* < 0.001 and overall musculoskeletal injuries.). The use of non-ridden training modalities was protective for dorsal metacarpal disease (HR 0.89, 95% CI 0.81, 0.97, *p* = 0.008), but not musculoskeletal injuries overall. The male sex increased the hazard of DMD compared to females (HR 2.58, 95% CI 1.20, 5.56, *p* = 0.02), but not MSI overall. In summary, the hazard of musculoskeletal injury is greatest for 2-year-old horses that are born from uniparous mares, commence training at a later age, are in their first training preparation, have undertaken little high-speed exercise or had limited rest before their training preparation. The hazard of dorsal metacarpal disease is greatest for 2-year-old horses that are males, commence training at a later age, are in their first training preparation, have undertaken little high-speed exercise or had limited use of non-ridden training modalities. Close monitoring of these high-risk horses during their training program could substantially reduce the impact of MSI. Furthermore, an understanding of how training methodologies affect the hazard of MSI facilitates modification of training programs to mitigate the risk impact of injury. The strengths of this study include a large sample size, a well-defined study protocol and direct trainer interviews. The main limitation is the inherent susceptibility to survival bias.

## Introduction

Musculoskeletal injuries (MSI) remain a global problem for the Thoroughbred racing industry, resulting in serious injury and/or death of horses (1–4) and riders (5, 6). There is conflicting evidence regarding the effect of age on MSI and the risk of MSI is different between 2-year-old horses and older horses (7–11). The types of MSI are also different between 2-year-old and older horses, with dorsal metacarpal disease (DMD) the most common type reported in 2-year-old horses (12–15). The ideal time for racehorses to commence training is strongly debated, with limited supporting literature. Mason et al. (16) reported a relationship between unsoundness and open distal radial epiphyses, however, there has been no further research to support these findings. In contrast, a series of experimental studies found that early race training facilitated superior tissue adaptation and was beneficial (17–26). Furthermore, longevity of racing career and improved performance is associated with starting horses at 2-years of age (27, 28). As yet, there have been no prospective studies evaluating whether early race training is beneficial or harmful to immature horses.

There is a strong interest in developing training and management strategies to reduce the impact of MSI. It is highly likely that the risk factors for MSI in 2-year-old horses are different than those for older horses, due to commencing race training prior to skeletal maturity. Skeletal maturity is frequently estimated by closure of the distal radial physis, and this occurs at around 2-years of age (16, 29). However, many other factors apart from growth influence the occurrence of MSI. These include high-speed exercise (HSE), cardiovascular fitness, tissue loading, rest and tissue adaptation (23, 25, 29, 30). It is also likely that the risk factors may vary between types of MSI.

High-speed exercise is likely to be a principal risk factor for MSI, although there is conflicting evidence regarding the effect of HSE on MSI. Some studies report that HSE increases the risk of MSI (31–40), while others report that it decreases (8, 40–47) or does not affect (8, 48–50) the risk of MSI. Other studies report a non-linear effect of HSE on the risk of MSI, whereby the risk initially decreases with increasing exercise, plateaus, then increases again (8, 36, 45, 47). The effect of HSE on MSI will also vary with type of injury (9, 35, 45, 51, 52).

Many MSI cases and fatalities occur during training rather than during racing (31, 33, 51–54). Therefore, studies analysing only race day MSI will miss a large proportion of cases that occur during training. These studies will also not capture MSI cases that are not apparent on the day of racing and are discovered later (54, 55). By combining racing and training data the true effect of risk factors on MSI may be more accurately represented. Furthermore, modifications to reduce the impact of MSI are more readily implemented at the training level.

Thus, there is a need for further research to investigate the risk factors for 2-year-old horses, with a focus on the age that training commences and the training strategies that may affect the risk of MSI. There is also a need to be able to identify at-risk individuals, so that these horses may be closely monitored during their training and the appropriate interventions applied. We address these knowledge gaps through a prospective cohort study of 2-year-old Thoroughbred racehorses in training. Our aims were: (1) to use survival analysis to determine the hazard of MSI for a number of horse and training-related risk factors, through a causal approach to model building and (2) Determine whether these hazards and risk factors were different for DMD than for other types of MSI.

## Materials and Methods

### Recruitment of Participants

This study was performed concurrently with a study investigating the overall incidence and types of MSI in Thoroughbred racehorses of all ages in Queensland, Australia. The recruiting process has been described previously in more detail (15). Human (2017001248) and animal (SVS/384/17) ethics approvals were obtained from The University of Queensland Science Low and Negligible Risk Human Ethics Sub-committee and the University of Queensland Animal Ethics Units, respectively. Trainers from the Brisbane Racing Club (BRC) were invited to participate in this weekly prospective study. Recruitment of horses was performed by recruiting trainers and enrolling all the 2-year-old horses from their training stables.

### Study Design

A prospective cohort study was conducted between November 2017 and December 2018. This time period was considered to best represent the 2-year-old racing season, because the first 2-year-old race in Queensland is at the end of October (https://www.racingqueensland.com.au/racing-and-results/full-calendar/2017/12). Detailed injury, training and exercise data were collected through personal structured interviews with participating trainers or their forepersons. Structured personal interviews facilitated accurate and complete data collection. Details of the interview are described in Appendix A1 in Supplementary Material.

### Data Collection

#### Two-Year-Old Horses

A horse was defined as a “2-year-old” until 1 August of its third year of life. August first is the date where Thoroughbred horses in Australia officially increase 1-year in age regardless of their actual date of birth. This definition includes all racehorses 2-years of age and younger, as racehorses in Australia are usually <2-years when they commence race training. All 2-year-old horses under the care of each recruited, licenced trainer were enrolled. Trainers were not able to select which horses contributed data. Horses were identified by both the name registered with Racing Australia (https://www.racingaustralia.horse/RoR/AboutROR.aspx) and microchip number. Sex was recorded as female or male. Males included both entire and castrated horses as males were frequently castrated during the study and following castration status was not feasible. The dam age at the time of the enrolled horses' birth and the dam parities were obtained from the Australian Stud Book (https://www.studbook.org.au/default.aspx). Horses were censored on August 1, 2018, when they turned 3-years of age, if they left the trainer and at study completion, December 2018.

#### Musculoskeletal Injuries

Time to failure was recorded in weeks. Data was recorded for recurrent event analysis; whereby individual horses could have more than one failure recorded. A failure was defined as any MSI, incorporating either orthopaedic or soft tissue injuries which prevented the horse from training for at least 7 days. A 7 day period was chosen to be consistent with previous studies (9, 54). This definition included any MSI that occurred whilst the horse was in training, whether the actual injury occurred during a race, training or following an accident in the stable. Osteochondritis dissecans, cervical stenotic myelopathy and other developmental orthopaedic conditions were included if the horse was in training, sound and later developed a clinical lameness or gait abnormality that prevented them from training. Musculoskeletal injuries were diagnosed by a veterinarian to minimise measurement and ascertainment bias. Horses in the study were under the close care of racetrack veterinarians registered in Queensland.

#### Exercise Variables as Putative Risk Factors for Musculoskeletal Injury

The key exercise variables examined as putative risk factors for MSI included exposure to high-speed exercise, pre-training before each preparation, the number of the training preparation, the rest period before each preparation, exposure to non-ridden exercise modalities and exposure to low-speed exercise. Daily training information was collected at weekly intervals. A training preparation was defined as the uninterrupted period that a horse is actively participating in race training. Horses could have rest days during a training preparation, but the preparation was considered complete if there were seven or more consecutive days of rest.

##### Exposure to High-Speed Exercise

The following four measures of exposure to high-speed exercise were calculated for each week from the daily exercise history:

1. The total distance (kilometres) travelled at a gallop (>13 s/furlong; 15 m/s; 900 m/min; 55 km/h). This consisted of the combined distance of track gallops, jump-outs (non-official trials), official trials and races. The official trial and race data was cross-checked with the Racing Australia Online Database (https://racingaustralia.horse/home.aspx).
2. The total number of days exercised at a gallop.
3. The total distance exercised at three-quarter pace (15 s/furlong; 13 m/s; 800 m/min; 48 km/h).
4. The total number of days exercised at three-quarter pace.

The weekly totals were then added to provide the total cumulative distance or days for each training preparation. Data from each training preparation were entered in multiple record format, to provide a total cumulative exposure to each of these variables over the study duration.

##### Pre-training Before Each Preparation

This was defined as the time (in weeks) that a horse undertook ridden exercise at a facility other than the racetrack before the training preparation. This information was collected from the trainer or foreperson during the structured interview. Horses commencing their first racing preparation were considered to have had 3-weeks of ridden pre-training exercise, because pilot studies with industry experts revealed marked variation in the time taken for non-ridden education during the “breaking in” process due to unmeasured factors including the horse's temperament, weather, the breaker used and the demands on the breakers' services. Expert consensus was that 3-weeks accurately represented the actual ridden exercise undertaken prior to beginning the first training preparation.

##### Number of the Training Preparation

The sequential number of the current training preparation was also completed for each horse, with the first preparation beginning when the horse first entered race training. This was not necessarily the same as the number of training preparations that the horse had completed during the study. For example, if a horse had completed two training preparations before entering the study, the first number of the training preparation recorded for that horse would be three. When a horse entered the study, the age that the horse commenced race training and the number of training preparations that the horse had previously completed was obtained from the trainer or foreperson.

##### Rest Period Before Each Training PREPARATIONS

Horses had rest periods in between training preparations. The length of time that the horse was rested after completion of one training preparation, prior to commencing the next preparation, was calculated in weeks. Horses commencing their first racing preparation were considered to have had no rest before their first preparation, as they had not undertaken any race training exercise.

##### Exposure to Non-ridden Exercise Modalities

The total number of days that the horse was exercised using non-ridden modalities was determined for each training preparation from the daily exercise data. This included walking exercise, and exercise using a water-walker, swimming pool or treadmill. Walking was defined as when horses were only exercised on the walking machine or led by hand. This did not include warm-up exercise on the walker prior to exercise on the racetrack, nor exercise on the walker in the afternoon in addition to morning exercise at the racetrack. Water-walkers were defined as walking machines in a shallow swimming pool, with the water up to approximately the level of the horses' chest. Treadmills were defined as stationary exercise machines with continuous belts that facilitate exercise at low or high speeds with or without an incline.

##### Exposure to Low-Speed Exercise

The total number of days that the horse was exercised at low-speed (slower than 15 s/furlongs; 13 m/s; 800 m/min; 48 km/h) was determined for each training preparation from the daily exercise data.

#### Power Calculations

Power calculations were based on the findings of a previous study (35), which reported a hazard ratio of 2.7 (95%CI 1.87–3.89) for every furlong increase in high-speed exercise distance. Sample size was estimated using the power module in Stata for cox regression, with the event of interest defined as involuntary spell of ≥7 days duration associated with a musculoskeletal injury. A sample size of 400 was sufficient to achieve 80% statistical power (alpha=0.05) for detection of a hazard ratio of 1.5 or more and with an expectation that 50% of the study population would develop the event during the study period with the remainder (50%) being right censored at the end of the study period.

#### Data Analysis

##### Causal Approach to Model Building

A causal approach (56), was used to inform parameterization of statistical models, incorporating published information and expert opinion on putative causal factors. This causal approach has advantages over rule-based methods of statistical model building and is becoming more widely used in analytical epidemiological studies (56–62). In particular, a causal approach enables consistent estimation of total or direct effects (as desired) and avoids stratification or collider bias, which results from inappropriate adjustment of variables and can lead to biassed estimates (56–62).

##### Explanatory Variables and Development of Directed Acyclic Graph

**Figure 1:**
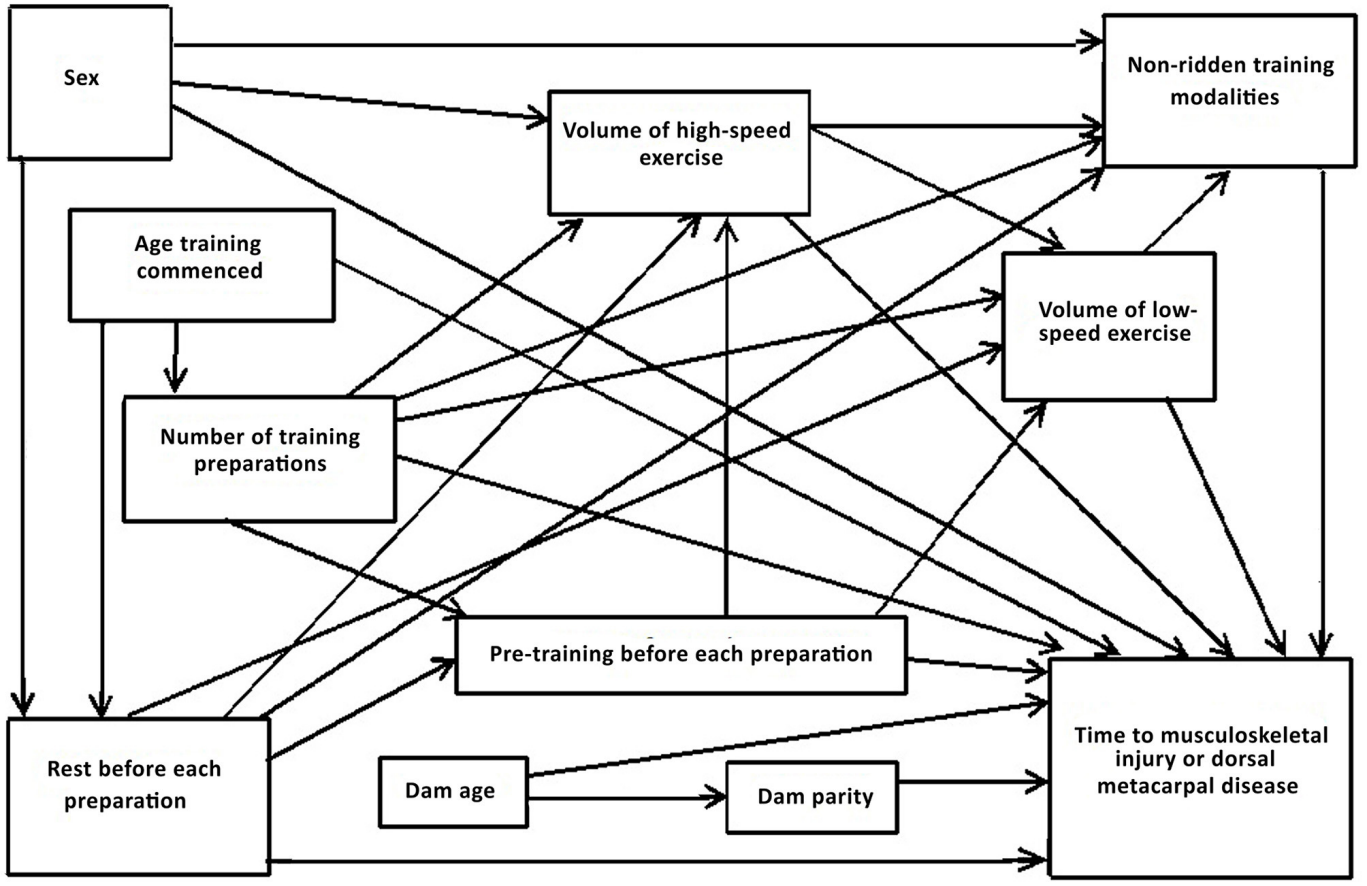
Directed acyclic graph depicting the postulated causal associations between putative risk factors for the time to musculoskeletal injury and dorsal metacarpal disease in 2-year-old Thoroughbred racehorses.

Directed acyclic graphs demonstrate the causal inter-relationships between explanatory variables (risk factors) as well as the causal associations of risk factors with an outcome variable (56, 57, 62). The measured explanatory variables were selected following discussion between epidemiologists and statisticians, veterinary surgeons and clinicians and industry experts. Discussions focused on identifying relevant putative causal variables for measurement; whether there were potentially important unmeasured variables; and the presence, absence and direction of each possible causal link between variables. Conclusions were based on scientific evidence where available and expert opinion if scientific literature was not available (Table 1). Potential biologically plausible interactions were also considered from the directed acyclic graphs (75). The resulting directed acyclic graph is presented in Figure 1.

**Table 1:** The causal relationships between explanatory variables (risk factors) for the time to musculoskeletal injuries and supporting evidence for these relationships.

**Variable**	**Direct causal effects on**	**Nature of effect**	**References**
Exposure to high-speed exercise	Non-ridden modalities	Increased used of non-ridden modalities when low volumes high-speed exercise required- pre-training and rehabilitation	(63)
	Exposure to low-speed exercise	Reduced low speed exercise on days fast work undertaken	^*^
	Time to MSI	Reduced MSI in preparations which had a race start	(45)
	Time to MSI	Reduced MSI with increased volume high-speed exercise	(8, 40–47)
	Time to MSI	Increasing cumulative racing distances were associated with an initial reduction in the odds of MSI that then levelled out and increased again as distance continued to increase	(8, 36, 45, 47)
	Time to MSI	Increased MSI with increased distance high-speed exercise	(14, 31–40, 64)
Pre-training before each preparation	Exposure to high-speed exercise	Increased exposure to high-speed exercise with longer pre-training duration	^*^
	Exposure to low-speed exercise	Lower volume low-speed exercise when longer pre-training duration	^*^
	Time to MSI	Lower risk of MSI with longer pre-training duration	^*^
Number of training preparations	Exposure to high-speed exercise	Increased rate of starts with increasing number of preparations	(64)
	Exposure to high-speed exercise	Increased high-speed exercise volume with increasing number of training preparations	^*^
	Pre-training before each preparation	Less pre-training is required when horse has had fewer training preparations as track education prioritised and pre-training required for fitness when older rather than education	^*^
	Exposure to low-speed exercise	Increased low-speed exercise volume with fewer number of training preparations	^*^
	Time to MSI	Reduced odds of MSI as training preparation number increases	(45)
	Non-ridden exercise modalities	Increased use of non-ridden modalities with increased number of training preparations	^*^
Age training commenced	Number of training preparations	The earlier the age training commences the higher potential number of training preparations as a 2-year-old	^*^
	Rest before each preparation	Horses starting at a younger age are given less rest before the next training preparation as they have been identified as 2-year-old types and are more likely to cope with exercise intensity and do not require same recovery time	^*^
	Time to MSI	Early race training facilitates superior tissue adaptation and could reduce risk MSI	(17–25)
Rest before each preparation	Pre-training before each preparation	More pre-training is required after a longer rest period	^*^
	Exposure to high-speed exercise	Larger high-speed exercise volumes are undertaken after shorter rest periods	^*^
	Non-ridden exercise modalities	Higher non-ridden exercise volume when longer rest periods	^*^
	Time to MSI	Increased rest increases the risk of MSI due to greater osteoclastic activity weakening bone structure	(65–67)
Non-ridden exercise modalities	Time to MSI	Decrease the incidence of MSI	(68)
	Time to MSI	Mechanical walking increased the risk MSI	(69)
Exposure to low-speed exercise	Non-ridden exercise modalities	Increased used of non-ridden modalities to replace low-speed exercise required- pre-training and rehabilitation	(39, 63)
	Time to MSI	Increased volume low-speed exercise increases risk MSI	(35)
Sex	Time to MSI	Males increased risk of MSI	(49, 50, 64, 70–73)
	Rest before each preparation	Males spent more time training and less time spelling	(64)
	Exposure to high-speed exercise	Females increased rate of race starts than males	(64)
	Non-ridden exercise modalities	Increased used of non-ridden modalities in females	(63)
Dam age	Dam parity	Dam parity is limited by the age of the dam	(74)
	Time to MSI	MSI decreased with increasing dam age	(74)
Dam parity	Time to MSI	First foals lower MSI than subsequent foals	(74)

* *Consensus from panel of experts in the field when scientific literature was not available*.

Minimum sufficient adjustment sets (the minimum sets of covariates that, when adjusted for, blocked all the back-door paths between the exposure and the outcome) were then determined from the directed acyclic graphs using Dagitty®, a graphical tool to estimate the total effects of each explanatory variable on each outcome of interest (56, 57, 62). The series of models and minimum sufficient adjustment sets are described in Table 2. There were no interactions that were considered appropriate for inclusion in the models.

**Table 2:** The series of models and associated minimum sufficient adjustment sets used to estimate the total effects of each putative risk factor on musculoskeletal injuries and dorsal metacarpal disease.

**Model**	**Putative risk factor**	**Minimum sufficient adjustment set**
1	Exposure to high-speed exercise	Number of training preparations
		Age training commenced
		Rest before each preparation
		Sex
		Pre-training before each preparation
2	Pre-training before each preparation	Number of training preparations
		Age training commenced
		Rest before each preparation
		Sex
3	Number of training preparations	Age training commenced
4	Age training commenced	Nil
5	Rest before each preparation	Age training commenced
		Sex
6	Non-ridden exercise modalities	Exposure to high-speed exercise
		Number of training preparations
		Age training commenced
		Rest before each preparation
		Sex
		Pre-training before each preparation
		Exposure to low-speed exercise
7	Exposure to low-speed exercise	Number of training preparations
		Age training commenced
		Rest before each preparation
		Sex
		Pre-training before each preparation
		Exposure to high-speed exercise
8	Sex	Nil
9	Dam age	Nil
10	Dam parity	Dam age

##### Statistical Analysis

Data were analysed using Stata 15.1® (Statacorp, College Station, TX, USA). The analysis time was the study period, which represents the 2-year-old racing season in Queensland. A multiple record format was used with horses entering and exiting the study according to training preparations and rest periods. A multiple record format also enables data to be collected and analysed for recurrent events and using time-varying covariates. Time-varying explanatory covariates can change in an inconsistent or unpredictable manner throughout the study period, which best represents the pattern of 2-year-old training methodologies (76). The explanatory variables that we analysed as time-varying covariates were exposure to high-speed exercise, the pre-training before each preparation, the number of the training preparation, the rest before each preparation, the use of non-ridden exercise modalities and the exposure to low-speed exercise. The age that training commenced, sex, dam age and dam parity were not analysed as time-varying covariates, as these variables did not change throughout the study period.

Descriptive statistics were reported for all failures because there were insufficient recurrent events for statistical analysis. Explanatory variables were summarised, stratified according to injury status. A Cox proportional hazards or Weibull regression model was run for each putative risk factor of interest with all variables in the minimum sufficient adjustment set required to estimate the total effect of that variable included in the model.

For Cox proportional hazards analysis, a clustered model was used to adjust for differences between trainers (14, 39, 77). The scale of continuous variables was examined using martingale residuals (77). Once the linear relationship to the log hazard was confirmed, variables were centred to the mean. The proportional hazards assumption was checked using scaled Schoenfeld residuals plotted over time (77). Goodness of fit was confirmed by plotting the Cox Snell residuals as failure times against the Nelson Aalen cumulative hazard function (77). Potential influential observations were checked by plotting Df-Beta residuals against time (77).

When the proportional hazards assumption was not met, or the goodness of fit was poor, a parametric accelerated failure time model was used. The appropriate accelerated failure time model was selected by firstly fitting a Weibull regression model because this model has a shaping parameter which allows the hazard to vary over time (77). The Wald test of the significance of the shaping parameter was evaluated to determine whether this model was more appropriate than the Exponential model, which has a constant hazard over time (77). Goodness of fit was evaluated by plotting the predicted estimates of the cumulative hazard against the Weibull model estimate of the cumulative hazard (77).

All variables were analysed as continuous variables, apart from sex, which was categorised into males and females. Polynomial terms (squared and cubic) for high-speed exercise variables were also tested in the models. Akaike's Information Criteria (AIC) and Bayesian Information Criteria (BIC) were used to compare models with and without polynomial terms. Significance was set at α = 0.05 for all statistical tests. Risk estimates were presented as hazard ratios for the time to MSI. Hazard ratios for estimates of interest represent the rate of risk at any point in time for each unit increase above the mean of continuous variables compared to the baseline hazard. Hazard ratios of <1 are “protective,” and hazard ratios >1 are “harmful.” For example, a hazard ratio of 0.8 can be interpreted as a 20% reduction in the rate of risk for every unit increase of the hypothesised risk factor, and conversely a hazard ratio of 1.2 can be interpreted as a 20% increase in the rate of risk for each unit increase of the hypothesised risk factor.

The hazards of MSI and DMD over the study period were depicted graphically for statistically significant explanatory variables as cumulative hazard functions for continuous variables. The default cumulative hazard function determined from the Weibull and Cox models represents the cumulative hazard at specified values of the main explanatory variable and the mean values of all continuous adjusting variables (77). A Kaplan Meier Curve was presented for the effect of sex. The specified values depicted for explanatory variables in our models were the 25th, 50th, and 75th percentiles because the median and interquartile range is the most appropriate way to present non-parametric data (59). Sensitivity analyses were conducted for high-speed exercise variables within strata of the age that training commenced and the stage of training, due to the possibility of survivor bias affecting the results. Analyses were then repeated for all models with the outcome defined as failure due to DMD.

## Results

The trainers who participated in this study also contributed to a concurrent study of musculoskeletal injuries and trainer characteristics have been described in detail previously (15). Briefly, 27 out of 40 eligible trainers (68%) agreed to participate. Data were collected every week for 56-weeks from November 2017 to December 2018 for 26/27 (96%) of trainers. One trainer did not train any 2-year-old horses for the study duration. Another trainer only contributed 6 months of data before retiring from training. Trainers provided exercise data for 535 2-year-old horses, who completed 1,258 training preparations over 7,512-weeks. Individual trainers had between 1 and 43 (median 14, IQR 6, 23) 2-year-old horses in training.

### Descriptive Statistics for Recurrent Failures

**Figure 2:**
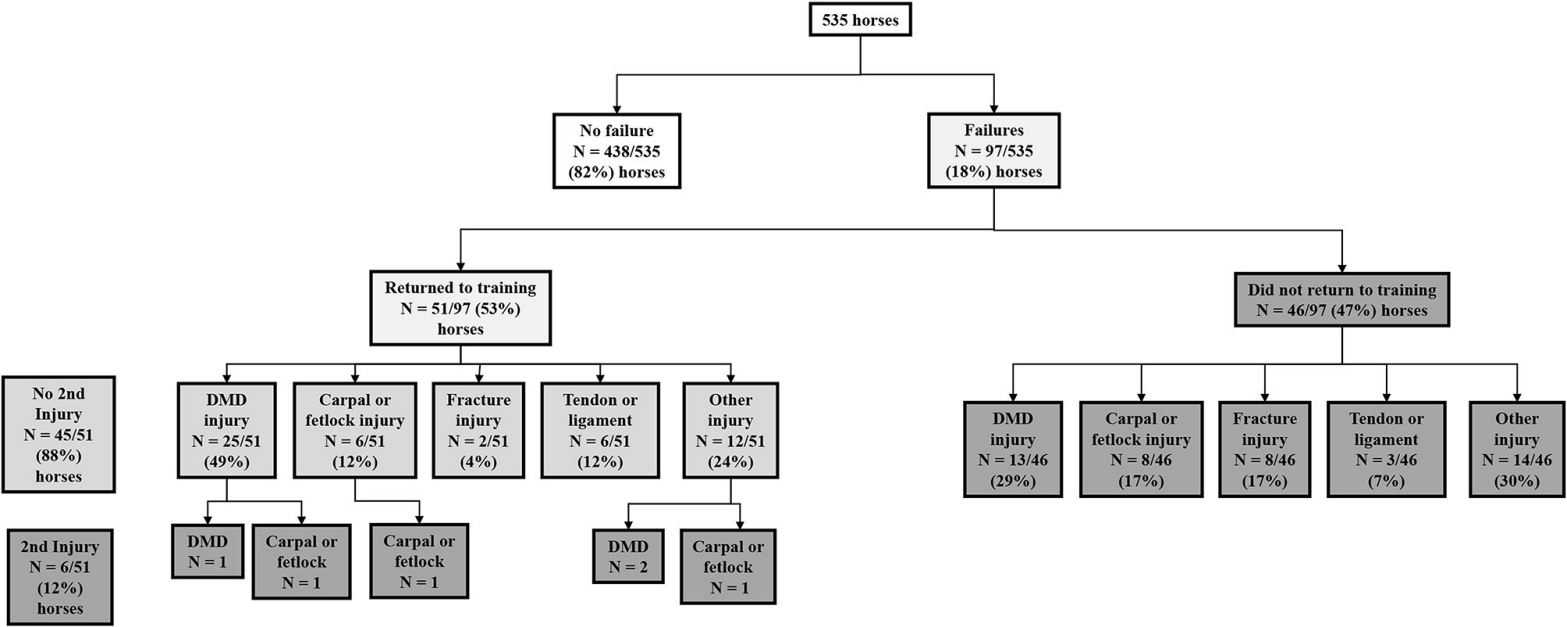
The flow of horses and injuries through the study investigating time to musculoskeletal injury in 535 2-year-old racehorses in training in Queensland, Australia.

We recruited a total of 535 2-year-old horses which provided exercise data for 1,258 training preparations over 7,512-weeks. There were 103 failures occurring in 97 horses. Of the 97 horses with a first failure event, 51/97 (53%) returned to the study after injury while they were still eligible at 2-years of age. No further failure was experienced in 45/51 (88%) of these horses, that returned to training for a mean period of 12-weeks, prior to being censored when turning 3-years of age, or at study completion. However, 6/51 horses (12%) returned to training for a mean of 11-weeks before sustaining a second failure. No horses sustained a third failure. The flow of horses and injuries through the study is presented in Figure 2.

### Failure Events Due to Musculoskeletal Injuries

Data were analysed as single-failure-per-subject rather than as recurrent events, because there were too few recurrent events recorded (*n* = 6) (77, 78). The 97 single-failure events occurred among the 535 horses over 1, 206 training preparations. The 97 single-failure events exceeded the minimum number for the predictor variables in all models (78). The survival function decreased to 75% at 17-weeks. Median survival (50%) was not reached by the conclusion of the 56-week study period.

### Failure Events Due to Dorsal Metacarpal Disease

There were 39 single-failure events, which occurred among 477 horses over 936 training preparations. The probability of survival did not decrease to 75% during the study period, thus median survival times are not presented.

### Risk Factors for Musculoskeletal Injury

The putative risk factors, stratified by injury status are presented in Table 3. Weibull regression models were used to evaluate the effect of exposure to high-speed exercise, pre-training before each training preparation, the rest before the training preparation, non-ridden exercise modalities and exposure to low-speed exercise on the time to failure. Cox proportional hazards regression models were used to evaluate the number of the training preparation, the age that training commenced, sex, dam age and dam parity on the time to failure. The results of all analyses are summarised in Table 4.

**Table 3:** The putative risk factors stratified by injury status in 535 2-year-old Thoroughbred racehorses in Queensland, Australia.

**Putative risk factor**	**Horses with injury (*N* = 97)**	**Horses without injury (*N* = 438)**
	**Median (IQR)**	
**Exposure to high-speed exercise**		
Total cumulative kilometres of gallop	1.6 (0.6, 3.6)	3.1 (0.4, 8.0)
Total cumulative days of gallop	5 (2, 8)	8 (2,16)
Total cumulative kilometres of three-quarter pace	3.0 (1.6, 5.2)	5 (2.2, 9.8)
Total cumulative days of three-quarter pace	7 (3, 11)	11 (5, 20)
**Pre-training before each preparation (weeks)**	3 (0, 3)	2 (0, 3)
**Number of training preparations**	2 (1, 2)	2 (1, 3)
**Age training commenced**	20 (18, 22)	20 (18, 22)
**Rest before each preparation (weeks)**	6 (3.5, 8)	7 (4, 9)
**Total cumulative days using non-ridden exercise modalities**	0 (0, 6)	1 (0, 14)
**Total cumulative days low-speed exercise**	29 (18, 45)	47 (27, 75)
**Sex**		
Males *N* (%)	55 (57)	206 (47)
Females *N* (%)	42 (43)	232 (53)
**Dam age**	9 (7, 11)	10 (8, 13)
**Dam parity**	3 (2, 4)	4 (2, 6)

**Table 4:** The series of univariable and multivariable models used to estimate the total effects of each putative risk factor on musculoskeletal injuries and dorsal metacarpal disease in 2-year-old Thoroughbred racehorses in Queensland, Australia.

**Putative risk factor**	**Musculoskeletal injuries overall**				**Dorsal metacarpal disease**			
	**Univariable**		**Adjusted**		**Univariable**		**Adjusted**	
	**Hazard ratio (95% CI)**	***P*-value**	**Hazard ratio (95% CI)**	***P*-value**	**Hazard ratio (95% CI)**	***P*-value**	**Hazard ratio (95% CI)**	***P*-value**
**Exposure to high-speed exercise**								
Total cumulative distance galloped (km)	0.84 (0.80, 0.88)	<0.001	0.89 (0.84, 0.94)	<0.001	0.79 (0.74, 0.84)	<0.001	0.89 (0.82, 0.97)	0.001
Total cumulative distance three-quarter pace (km)	0.88 (0.83, 0.93)	<0.001	0.94 (0.90, 0.99)	0.04	0.87 (0.80, 0.93)	<0.001	0.96 (0.90, 1.03)	0.25
Total cumulative days galloped	0.92 (0.89, 0.95)	<0.001	0.95 (0.92, 0.98)	0.001	0.89 (0.86, 0.93)	<0.001	0.96 (0.92, 0.99)	0.03
Total cumulative days three-quarter pace	0.93 (0.90, 0.95)	<0.001	0.93 (0.93, 0.99)	0.01	0.92 (0.88, 0.96)	<0.001	0.98 (0.94, 1.02)	0.26
Pre-training before each preparation (weeks)	1.24 (1.07, 1.44)	0.01	1.05 (0.82, 1.34)	0.71	1.39 (1.14, 1.70)	0.001	1.13 (0.81, 1.59)	0.47
Number of the training preparation	0.55 (0.46, 0.66)	<0.001	0.58 (0.50, 0.67)	<0.001	0.40 (0.27, 0.58)	<0.001	0.43 (0.30, 0.61)	<0.001
Age training commenced (months)	1.13 (1.06, 1.19)	<0.001	n/a	n/a	1.17 (1.07, 1.28)	<0.001	n/a	n/a
Rest before each preparation (weeks)	0.87 (0.80, 0.95)	0.002	0.89 (0.83, 0.96)	0.003	0.89 (0.77, 1.03)	0.12	0.91 (0.80, 1.03)	0.12
Non-ridden exercise modalities (days)	0.98 (0.96, 0.98)	<0.001	0.98 (0.96, 1.00)	0.05	0.89 (0.85, 0.94)	<0.001	0.89 (0.81, 0.97)	0.008
Exposure to low-speed exercise (days)			0.99 (0.98, 1.00)	0.16	0.97 (0.96, 0.99)	<0.001	0.99 (0.97, 1.00)	0.25
**Sex**								
Females	Reference				Reference			
Males	1.22 (0.89, 1.66)	0.07	n/a	n/a	2.58 (1.20, 5.56)	0.02	n/a	n/a
Dam age (years)	0.96 (0.91, 1.00)	0.06	n/a	n/a	0.98 (0.88, 1.09)	0.70	n/a	n/a
Dam parity	0.90 (0.84, 0.97)	0.01	0.86 (0.77, 0.97)	0.01	0.92 (0.80, 1.06)	0.26	0.84 (0.64, 1.11)	0.21

*n/a, not applicable*.

#### Factors That Were Protective Against Musculoskeletal Injury

**Figure 3:**
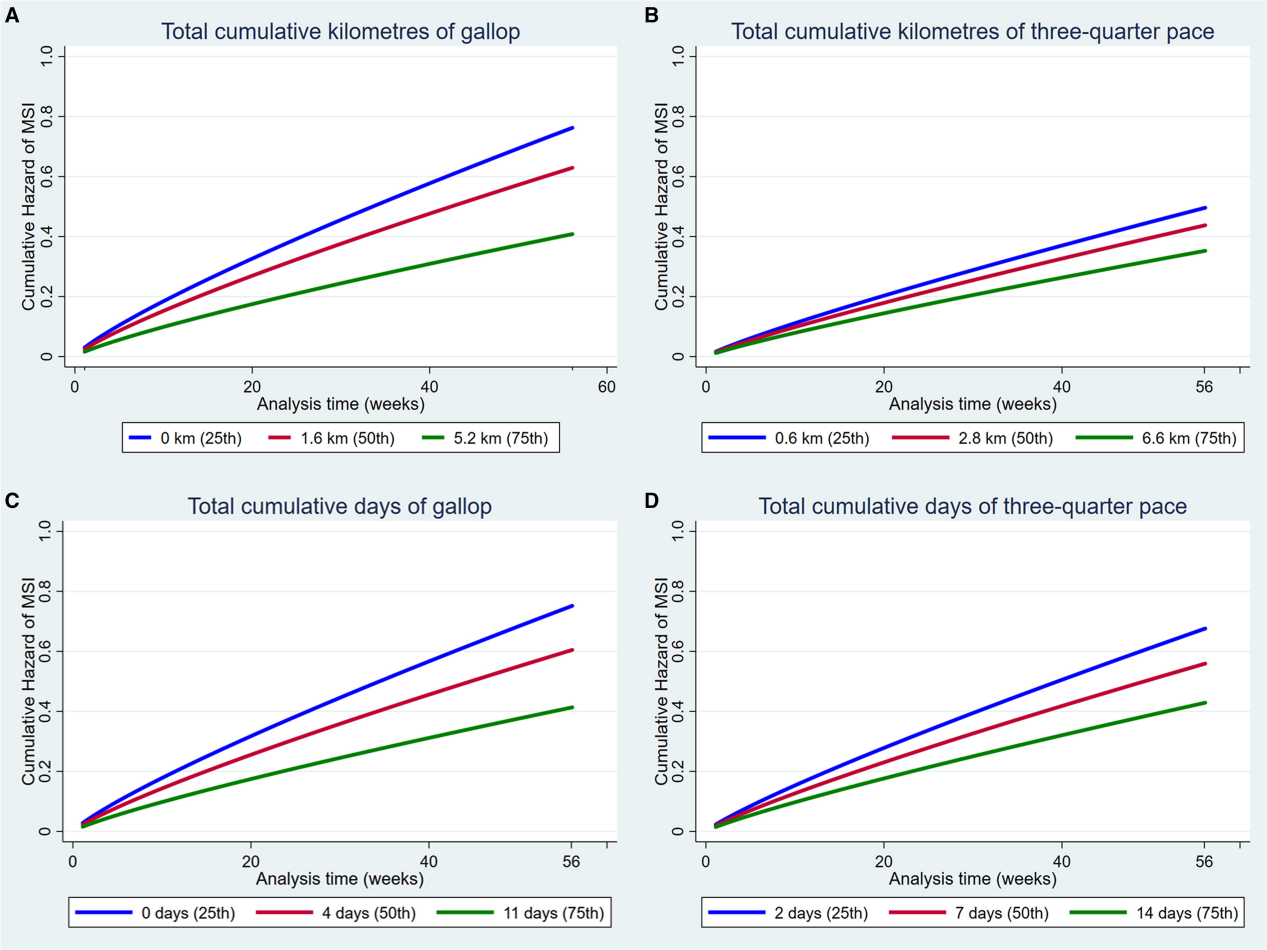
Cumulative hazard functions of musculoskeletal injury for the 56-week study period. Hazard functions are derived from Weibull multivariable regression models for four measures of the exposure to high-speed exercise: **(A)** Total cumulative kilometres of gallop, **(B)** Total cumulative kilometres of three-quarter pace, **(C)** Total cumulative days of gallop and **(D)** Total cumulative days of three-quarter pace. Graphs of the models are presented at the 25th, 50th, and 75th percentiles of each high-speed exercise measure.

Increased exposure to all four measures of high-speed exercise reduced the hazard of MSI over the duration of the study (Figure 3). Incorporation of squared and cubic polynomial terms for high-speed exercise did not decrease the AIC or BIC. Sensitivity analyses were then performed within strata of the age that training commenced, and the coefficients did not change between strata. However, when analyses were repeated within strata according to the length of time that horses had been in training, the risk of injury decreased with increasing time in training (Early stage: HR = 1.14, 95% CI 0.95, 0.99, *p* = 0.12; Mid stage: HR = 0.99, 95% CI 0.97, 1.02, *p* = 0.86; Late stage: HR = 0.97 95% CI 0.95, 0.99 *p* = 0.02).

**Figure 4:**
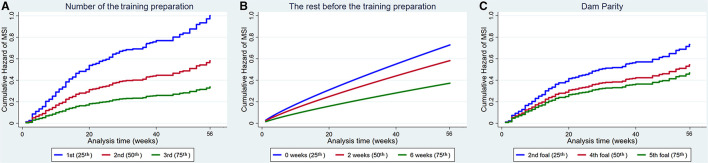
Cumulative hazard functions of musculoskeletal injury for the 56-week study period. Hazard functions are derived from Cox proportional hazard models **(A,C)** and Weibull **(B)** multivariable regression models for **(A)** The number of the training preparation, **(B)** The rest before the training preparation and **(C)** Dam parity. Graphs of the models are presented at the 25th, 50th, and 75^th^ percentiles of each explanatory variable.

Increased number of the training preparation, rest before the training preparation and dam parity also reduced the hazard of MSI (Figure 4).

#### Factors That Were Harmful for Musculoskeletal Injury

**Figure 5:**
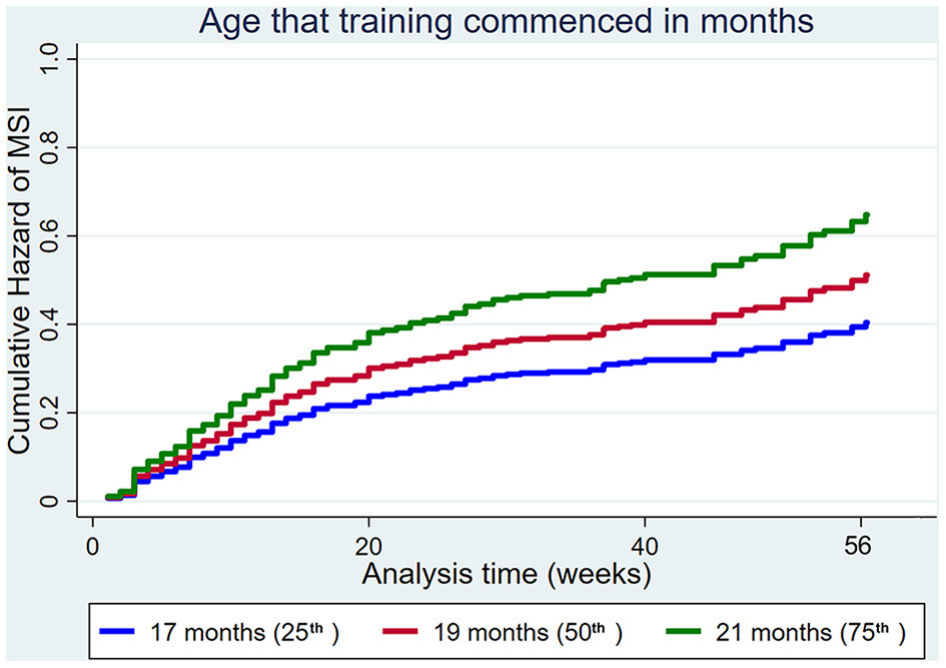
Cumulative hazard functions of musculoskeletal injury for the 56-week study period. Hazard functions are derived from Cox proportional hazard regression models for the age that training commenced. Graphs of the models are presented at the 25th, 50th, and 75th percentiles of each explanatory variable.

The age that training commenced increased the hazard of MSI (Figure 5).

#### Factors That Had No Apparent Association With the Risk of Musculoskeletal Injury

There was no evidence of a moderate or large effect of the amount of pre-training before each training preparation (HR 1.05, 95% CI 0.82, 1.34, *p* = 0.71) or the exposure to low-speed exercise (HR 0.99, 95% CI 0.98, 1.00, *p* = 0.16) on the hazard of MSI. There was weak evidence that the use of non-ridden training modalities (HR 0.98, 95% CI 0.96, 1.00, *p* = 0.05) and dam age (HR 0.96, 95% CI 0.91, 1.00, *p* = 0.06) affected the hazard of MSI, although this was not significant. The point estimate for the hazard of sex on MSI was too imprecise to enable a conclusion to be reached (HR males 1.22, 95% CI 0.89, 1.66, *p* = 0.07).

### Risk Factors for Dorsal Metacarpal Disease

#### Factors That Were Protective Against Dorsal Metacarpal Disease and Musculoskeletal Injuries Overall

**Figure 6:**
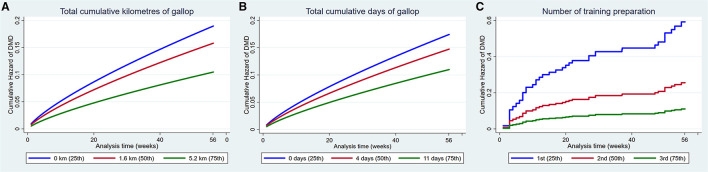
Cumulative hazard functions of dorsal metacarpal disease for the 56-week study period. Hazard functions are derived from Weibull **(A,B)** and Cox proportional hazard regression models **(C)** multivariable regression models for **(A)** The total cumulative kilometres of gallop exercise, **(B)** The total cumulative days of gallop exercise and **(C)** The number of the training preparation. Graphs of the models are presented at the 25th, 50th, and 75th percentiles of each explanatory variable.

Increased total cumulative distance (HR 0.89, 95% CI 0.82, 0.97, *p* = 0.001) and total cumulative days exercised as a gallop (HR 0.96, 95% CI 0.92, 0.99, *p* = 0.03) and the number of the training preparations (HR 0.44, 95% CI 0.32, 0.62, *p* < 0.001) reduced the hazard of DMD (Figure 6).

#### Factors That Were Harmful for Dorsal Metacarpal Disease and Musculoskeletal Injuries Overall

**Figure 7:**
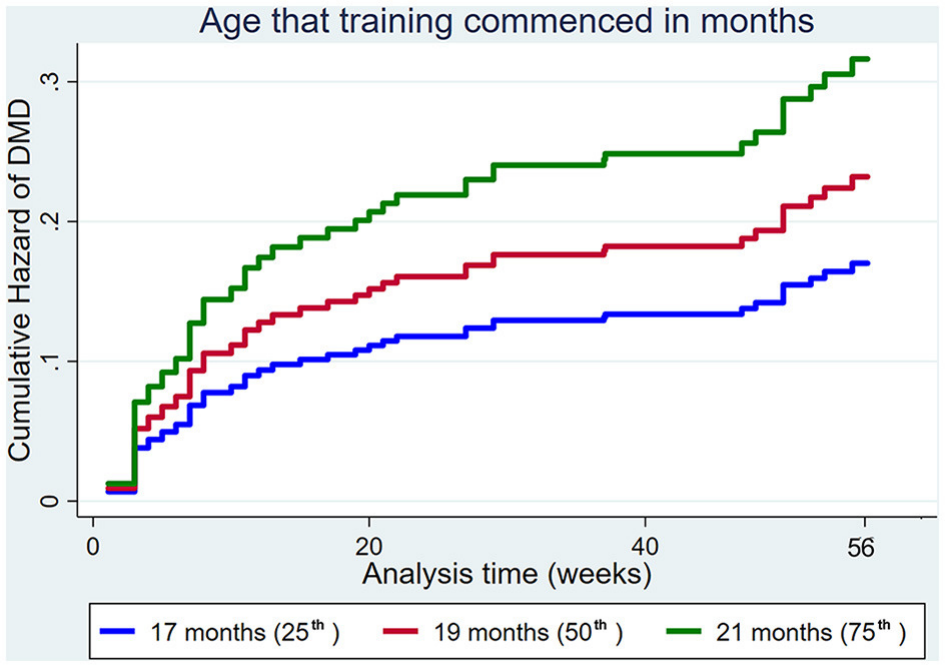
Cumulative hazard functions of dorsal metacarpal disease for the 56-week study period. Hazard functions are derived from Cox proportional hazard regression models for the age that training commenced. Graphs of the models are presented at the 25th, 50th, and 75th percentiles of each explanatory variable.

Increasing the age that training commenced increased the hazard of DMD (Figure 7).

#### Factors That Had No Significant Association With the Risk of Dorsal Metacarpal Disease or Musculoskeletal Injury

There was no evidence of a moderate or large effect of the exposure to low-speed exercise (HR 0.99, 95% CI 0.97, 1.00, *p* = 0.25) on the hazard of DMD. The point estimates for the amount of pre-training before each preparation (HR 1.13, 95% CI 0.81, 1.59, *p* = 0.47) and dam age (HR 0.98, 95% CI 0.88, 1.09, *p* = 0.70) were too imprecise to enable conclusions to be reached.

#### Factors That Were Protective Against Dorsal Metacarpal Disease but Not Musculoskeletal Injuries Overall

**Figure 8:**
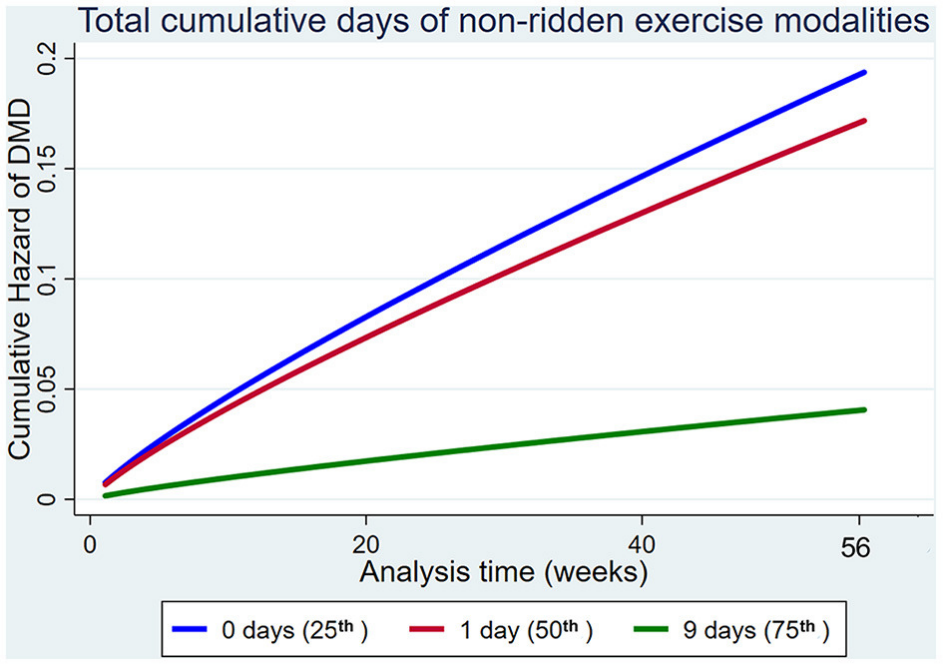
Cumulative hazard functions of dorsal metacarpal disease for the 56-week study period. Hazard functions are derived from Weibull regression models for the total cumulative days of non-ridden exercise. Graphs of the models are presented at the 25th, 50th, and 75th percentiles of each explanatory variable.

Increased use of non-ridden training modalities decreased the hazard of DMD, but not MSI overall (Figure 8).

#### Factors That Were Harmful for Dorsal Metacarpal Disease but Not Musculoskeletal Injuries Overall

**Figure 9:**
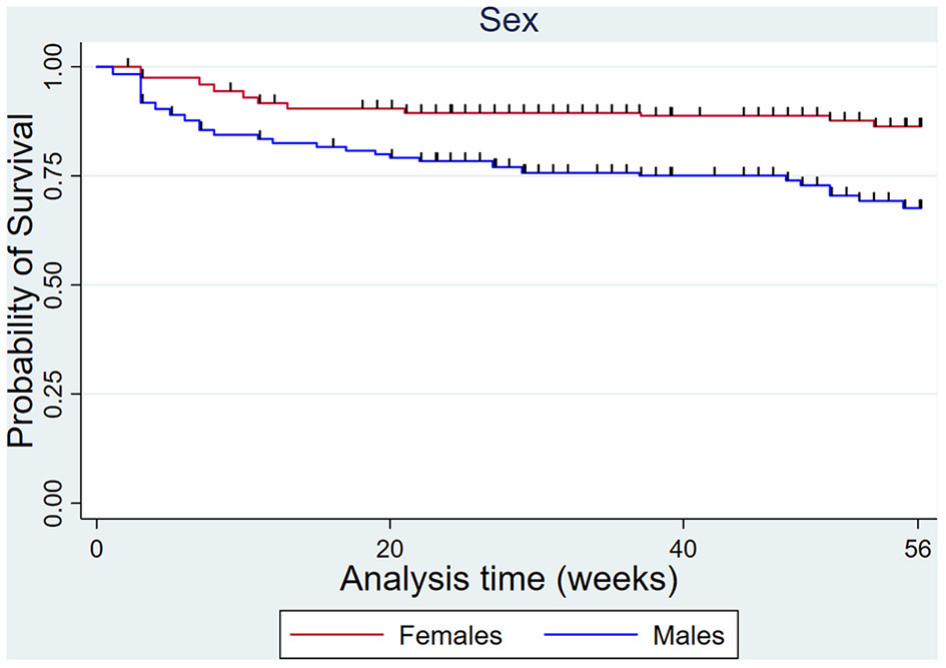
Kaplan Meier survival curves for dorsal metacarpal disease in males and females over the 56-week study period.

The male sex increased the hazard of DMD compared to females, but not MSI overall (Figure 9).

#### Factors That Had No Apparent Association With the Risk of Dorsal Metacarpal Disease but Were Significant for Musculoskeletal Injuries Overall

There was no evidence of a moderate or large effect of the cumulative distance (HR 0.99, 95% CI 0.98, 1.01, *p* = 0.25) and cumulative days (HR 0.98, 95% CI 0.94, 1.02, *p* = 0.26) travelled at three-quarter pace on the hazard of DMD. The point estimates for the amount of rest before each preparation (HR 0.91, 95% CI 0.81, 1.03, *p* = 0.13) and dam parity (HR 0.84, 95% CI 0.64, 1.11, *p* = 0.23) were too imprecise to enable conclusions to be reached.

## Discussion

This paper used a causal approach to investigate the effect of detailed training methodologies and other putative risk factors on MSI and DMD. This causal approach has many advantages over the traditional rule-based method of statistical model building and is now becoming more widely used in human and veterinary analytical epidemiological studies (56–62). The most important advantages of the causal approach over the traditional rule-based method of model building are that the causal approach enables consistent estimation of total or direct effects (as desired) and avoids stratification or collider bias, which results from inappropriate adjustment of variables and can lead to biassed estimates (56–62).

Our finding that high-speed exercise exposure reduced the hazard of MSI based on all four measures evaluated and reduced the hazard of DMD for total cumulative distance and days of gallop is consistent with previous studies reporting a decreased risk of MSI with increasing high-speed exercise (8, 40–47). These findings are also biologically plausible because a level of high-speed exercise is required for bone (42, 65, 66, 79–83) and tendon or ligament (17, 19, 29, 62, 84–87) adaptation necessary to prevent injury. The protective effect of high-speed-exercise observed in this study suggests that the high-speed exercise volume undertaken was still within the range required for tissue adaptation, as increasing the high-speed exercise volume beyond the level required for adaptation increases the risk of MSI (8, 36, 45, 47). This may be due to selection bias, whereby those trainers that agreed to participate in the study may be more concerned about MSI than trainers that did not participate and were less likely to exercise their horses at a harmful level (59, 88). There is also likely to be an effect of survival bias, whereby those horses that are injured earlier are removed from the population before a large exposure to high-speed exercise is accumulated, and those remaining are at reduced risk of injury (59, 88). Our sensitivity analyses confirmed that survival bias was likely to be influencing the results of this study. Horses in the earlier stage of training were more likely to be injured than those in the mid or late stages of their training.

The hazard of all types of MSI and specifically DMD were reduced with increased number of training preparations within the study period. An increasing number of preparations may be due to a larger number of short training preparations, rather than a smaller number of long preparations, although this could not be evaluated using survival analysis, due to the right-censoring of data. We postulate that a higher number of shorter preparations in the early stages of training reduce the hazard of injury by providing sufficient stimulus to facilitate bone adaptation but alternating this with rest minimises fatigue and enables tissue repair before microdamage progresses. Providing short periods of rest in training programs has been recommended to minimise fatigue and injuries in military recruits (89) and elite ballet dancers (90). Survival bias may accentuate these findings (59, 88).

The age that training commenced also affected the hazard of MSI and DMD, with increasing age associated with a higher hazard of injury. This finding is consistent with a previous study reporting a higher hazard of DMD in horses that commenced training at 21 months and above older than (91). In contrast, another study reported that the age that training commenced did not affect the hazard of DMD (35). The difference in findings may be due to the case definition of DMD. The case definition of DMD in the study by Verheyen et al. (35) included all incident cases of DMD, regardless of the number of training days lost. The case definitions reported by Jackson et al. (91) and the current study were at least five and seven consecutive days lost to training, respectively.

An increased hazard of MSI and DMD associated with increasing age that training commenced is also biologically plausible. The tendons and ligaments of foals and young horses can adapt to exercise in response to the mechanical forces imposed, whereby the volume and cross-sectional area increases (17, 19, 29, 62, 84–87). However, the tendon structure is mature by 2-years of age, after which there is no further adaptation to exercise and training and tendon structural deterioration occurs synergistically with increasing age and exercise (17, 29, 70, 84–87, 92). It is plausible that commencing training at an increased age reduces the narrow opportunity for tissue adaptation. Similarly, commencing race training at a young age has been shown to improve cortical bone density and hyaline cartilage of the third carpal bone (21), third metacarpal and metatarsal bones (22) and condyle size (22). Positive effects of early race training on gait and kinematic parameters have also been reported (21, 23). It is important that the loads encountered during racing are experienced during training, to permit the functional adaptation required for protection against injury (42, 93, 94).

Increasing the amount of rest before the training preparation reduced the hazard of MSI. Whilst there are no other reports specifically describing the association between rest periods and MSI hazard, previous research has shown that when training has resumed before the formation phase of remodelling is complete, the hazard of injury is increased (67). This is due to reduced bone strength because osteoclastic activity has weakened the structure prior to the osteoblastic phase and deposition of replacement bone (65, 66).

Increased use of non-ridden training modalities reduced the hazard of DMD, but we failed to detect an effect on all MSI. The difference in significance between injury types may because the weight of the rider is a more important risk factor for DMD than other types of MSI. Unfortunately, we were not able to measure the weight of the riders. Other studies have also reported that non-ridden exercise may reduce the hazard of MSI (63, 69, 95).

Males were significantly more likely than females to develop DMD, although they were not at increased hazard of MSI overall. Other studies have also reported a higher risk of DMD in males (64). This finding is biologically plausible because males, particularly entire colts, differ in body composition from females being in general heavier and having proportionately greater muscle mass than females. They are more likely to overwork or misbehave during training, which increases the forces on the dorsal cortex of the third metacarpal bone. The dorsal metacarpal bone may be more susceptible to these increased forces than other bones and joints.

Increasing dam parity also reduced the overall hazard of MSI, although we failed to detect an effect for DMD. In contrast, the only other study investigating dam parity as a risk factor reported that the risk of fracture was lower for first foals (74). The difference in findings may be attributed to the analytical methods used. Verheyen et al. (74) used multivariable Poisson regression analysis, adjusted for high-speed exercise, whereas in the current study we used survival analysis, and the causal approach negated adjustment for high-speed exercise. Furthermore, Verheyen et al. (74) commented in their discussion that this finding was contrary to their hypothesis. It is biologically plausible that increasing dam parity could decrease the risk of MSI through increasing birthweight and, therefore, increasing volumetric bone mineral density. Multiparous mares are known to produce foals with heavier birthweight than primiparous mares (96). Heavier bodyweight is associated with a higher volumetric bone density in human children (97), although this information is not available for horses. Increased volumetric bone density is protective for MSI in humans (98) and horses (99, 100).

The main strength of this study was detailed, high quality data for a large number of risk factors, resulting from access to a l large number of the trainers through personal interviews. Personal interviews ensured that the data collected was both complete and accurate, rather than relying on trainers to complete standardised questionnaires (9, 101, 102). The prospective study design with weekly data collection minimised the inherent recall bias commonly associated with case-control studies (88). Using a causal approach also provides appropriate adjustment of variables in the statistical modelling approach (57, 58, 62).

The main limitation of this study is the inherent survival bias. Furthermore, this population of 2-year-old horses represents a subset of the Australian racing industry and our results may not be globally applicable. Furthermore, our findings are only applicable within the reported range of the exposure variables. Extrapolating findings beyond this measured range is inappropriate and potentially harmful.

## Conclusion

The overall hazard of MSI was reduced with increasing exposure to high-speed exercise, increased number of training preparations, rest before the training preparation and increasing dam parity. The hazard of MSI was increased with increasing age that training commenced. Thus, in this population, the hazard of musculoskeletal injury is greatest for a subset of 2-year-old horses that are born from uniparous mares, commence training at a later age, are in their first training preparation, have undertaken little high-speed exercise or had limited rest before their training preparation. The hazard of DMD was reduced with increasing exposure to high-speed exercise, increased number of training preparations and increased use of non-ridden exercise modalities, while DMD hazard was increased with increasing age that training commenced, and for males. Thus, in this population, the hazard of dorsal metacarpal disease is greatest for a subset of 2-year-old horses that are males, commence training at a later age, are in their first training preparation, have undertaken little high-speed exercise or had limited use of non-ridden training modalities. Close monitoring of these high-risk horses during their training program combined with appropriate intervention could substantially reduce the impact of MSI in 2-year-old Thoroughbred racehorses. Furthermore, an understanding of how training methodologies affect the hazard of MSI facilitates modification of training programs to mitigate the impact of injury.

## Data Availability Statement

The raw data supporting the conclusions of this article will be made available by the authors, without undue reservation.

## Ethics Statement

The studies involving human participants were reviewed and approved by the University of Queensland Science Low and Negligible Risk Human Ethics Sub-committee. The patients/participants provided their written informed consent to participate in this study. The animal study was reviewed and approved by University of Queensland Animal Ethics Units. Written informed consent was obtained from the owners for the participation of their animals in this study.

## Author Contributions

KC, RG, CP, NP, and BA contributed to conception of the study. KC collected data with assistance from EB. KC organised the database and wrote the first draught of the manuscript. KC performed the statistical analysis with guidance from AF, RG, TB, SW, and NP. All authors contributed to design of study in various ways, contributed to manuscript revision, read, and approved the submitted version.

## Conflict of Interest

The authors declare that the research was conducted in the absence of any commercial or financial relationships that could be construed as a potential conflict of interest.

## Publisher's Note

All claims expressed in this article are solely those of the authors and do not necessarily represent those of their affiliated organizations, or those of the publisher, the editors and the reviewers. Any product that may be evaluated in this article, or claim that may be made by its manufacturer, is not guaranteed or endorsed by the publisher.
