# Supplementary figures and images for: Survival Analysis of Training Methodologies and Other Risk Factors for Musculoskeletal Injury in 2-Year-Old Thoroughbred Racehorses in Queensland, Australia
Source: Front Vet Sci. 2021 Nov 2;8:698298. doi: 10.3389/fvets.2021.698298 (PMC8593238; doi:10.3389/fvets.2021.698298)

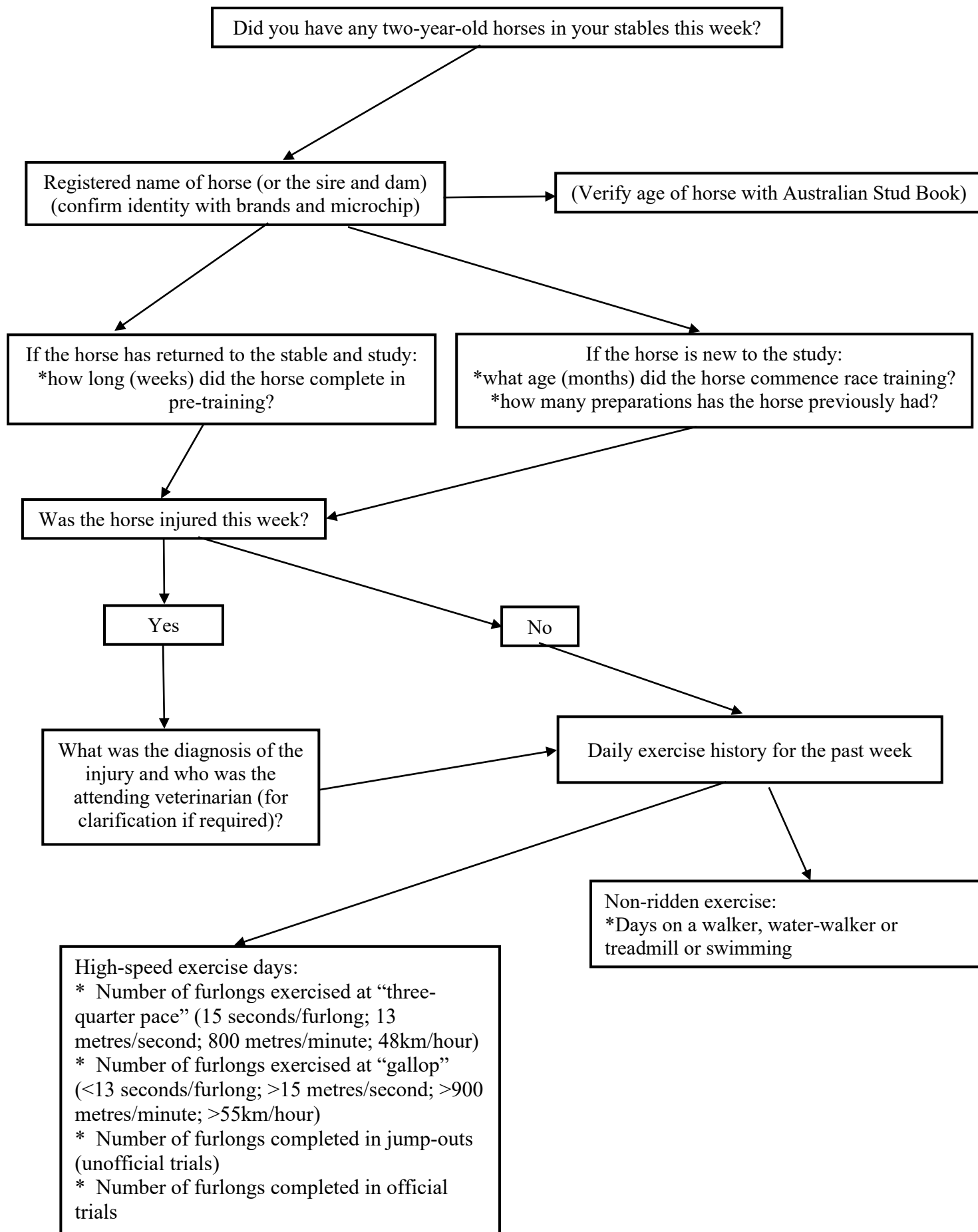

Supplement: Supplementary file 1 [file Data_Sheet_1.PDF]
